# Determinants of Self-Medication With Antibiotics in European and Anglo-Saxon Countries: A Systematic Review of the Literature

**DOI:** 10.3389/fpubh.2018.00370

**Published:** 2018-12-17

**Authors:** Dominique Lescure, John Paget, Francois Schellevis, Liset van Dijk

**Affiliations:** ^1^Erasmus MC, University Medical Center Rotterdam, Rotterdam, Netherlands; ^2^Department of Primary Care, Netherlands Institute for Health Services Research (NIVEL), Utrecht, Netherlands; ^3^Department of General Practice and Elderly Care Medicine, Netherlands Institute for Health Services Research (NIVEL), Utrecht, Netherlands; ^4^EMGO Institute for Health and Care Research, VU University Medical Center Amsterdam, Amsterdam, Netherlands

**Keywords:** non-prescription drugs, over-the-counter, anti-bacterial agents, motivation, explanation

## Abstract

**Background:** Self-medication with antibiotics, which comes in different forms [e.g., leftover or over-the-counter (OTC) use], contributes to antimicrobial resistance as it often happens in a non-prudent manner. In order to tackle this persistent public health problem, its drivers need to be known. The aim of this study was therefore to identify determinants of self-medication with antibiotics via a systematic literature review.

**Methods:** A comprehensive search on determinants of self-medication with antibiotics in the ambulatory care was conducted in PubMed, Scopus, and Embase for studies published between January 2000 and March 2017. There was no limit on the language nor on the type of study. The search was restricted to European and Anglo-Saxon countries. Pairs of reviewers independently screened the abstracts and full texts and performed a quality assessment.

**Results:** From the initial 664 abstracts, 54 publications that included 44 countries were retrieved of which most identified patient related determinants. Important determinants include storing antibiotics at home, poor access to healthcare, and having the intention to self-medicate. Healthcare professionals contribute to the practice of self-medication when catering for demanding and socially vulnerable patients. Healthcare system related determinants include dispensing antibiotics in whole packages and the lack of enforcement of medicine regulations. For some determinants (e.g., patients' age) contradictory results were found.

**Conclusion:** Self-medication with antibiotics is driven by a variety of determinants on the patient, healthcare professional, and system levels. Policy makers should recognise the complexity of self-medication in order to develop multifaceted interventions that target healthcare professionals and patients simultaneously.

## Introduction

Self-medication with antibiotics in the ambulatory care setting is a persistent and relevant public health problem in European and Anglo-Saxon countries. In the European Union the percentage of patients who self-medicated with antibiotics was estimated to be about 5% in 2009 and increased to 7% according to the latest estimations in 2016. The highest rates within the European Union (EU) can be found in southern European countries like Greece (20%), Romania (16%), and Cyprus (14%). In contrast, European countries like Sweden (2%) and Slovakia (3%) have the lowest rates of self-medication with antibiotics (1). When focusing more broader on the World Health Organization (WHO) Euro region and Anglo-Saxon countries, even higher rates have been found in countries like Russia (83.6%), Central America (19%), the former Yugoslav Republic of Macedonia (17.8%), and Latin America (14–26%) (2, 3). These numbers reveal that self-medication with antibiotics is not only a health problem for developing countries, but also for developed countries. As self-medication often happens in a non-appropriate way without medical guidance, it is an important contributor to antimicrobial resistance (4).

Community pharmacies that dispense antibiotics without a medical prescription are important stimulators for self-medication. In countries where the sale of over-the-counter (OTC) drugs is prohibited but not enforced, like in many Latin American countries, self-medication with antibiotics is common (5). Moreover, in the EU where OTC sales of antibiotics is illegal, it occurs frequently in some countries such as Spain or Greece (6–8). Besides OTC selling, other opportunities to obtain antibiotics without a prescription are buying antibiotics over the internet or the use of leftovers from family or friends or from previous courses. Overall, the use of leftovers was most prevalent in Southern, Northern and Western European countries whereas obtaining antibiotics at the pharmacy without a prescription was the major source for self-medication in Eastern European countries (9).

As self-medication with antibiotics is an alarming phenomenon which appears in different forms and can have negative public health effects, it is important to identify determinants that drive patients to use antibiotics without a medical prescription. When examining these determinants it is essential to use a broad perspective by focusing on multiple levels. Within the healthcare context determinants can be found at three levels: the patient level (e.g., the behaviour of individuals), the healthcare professional level (e.g., services provided by healthcare professionals), and the healthcare system level (e.g., legislation).

Various European and Anglo-Saxon studies have focused on determinants that are associated with self-medication (7, 9–11). Despite advanced healthcare systems and strict regulation in developed countries, it is striking self-medication with antibiotics exists and that patients find ways to self-medicate with antibiotics. This makes it even more important to gain insight in relevant determinants that explain self-medication in these countries. To our knowledge, there is no overview for European and Anglo-Saxon countries that distinguishes between the three mentioned levels and combines the available information of determinants on these levels. The aim of this study is therefore to identify determinants of self-medication with antibiotics in European and Anglo-Saxon countries from the scientific literature at three levels: the patient, healthcare professionals, and the healthcare system.

## Methods

### Search Strategy

A comprehensive and systematic literature search was conducted in the scientific electronic databases PubMed, Scopus, and Embase. We searched for studies published from January 2000 to March 2017 without a limitation on language or type of study. The search string included the following terms and their equivalents: (anti-bacterial agents OR drug resistance OR antibiotic) AND (non-prescription OR over-the-counter OR self-medication) AND (Europe OR Anglo-Saxon countries). European and Anglo-Saxon countries were defined as developed countries that share fundamental political ideologies like human rights and that make use of similar healthcare systems. From a geographically perspective, we defined Europe as being the WHO Euro area (which spans from Portugal in the west to the Russian Federation in the east), minus Israel. To ensure our search captured all relevant articles that are related to these countries, we added the individual country names to the search string. Self-medication was defined as actual self-medication and the intention to self-medicate (patients who intend to self-medicate in the future or who are in the possession of leftovers). The electronic searches were supplemented by manual searches of reference lists and citation tracking of articles that met our inclusion criteria.

### Selection Criteria

A study was selected if all of the following criteria were met: (1) it addresses determinants of self-medication with antibiotics within the geographical context of at least one EU Member State, the WHO Euro region countries or Anglo-Saxon countries; (2) it is an empirical study; (3) if self-medication includes other medicines the quantitative study has to incorporate a subgroup analysis for antibiotics. There was no specified criteria with regard to the methods used in the empirical studies, making this a systematic review with a mixed method approach in which both quantitative and qualitative studies were included. Articles were excluded if they focused on antibiotics in hospital settings, animals, or agriculture.

### Data Synthesis and Analysis

Firstly, the titles were screened by two authors (a combination of DL, JP, and LvD), followed by a screening of abstracts by the same authors. Disagreements were resolved by discussion between the two authors. Next, the first author (DL) extracted data from the selected publications using a digital data extraction form. Another author (LvD) checked the extracted data.

For each included study, the following information was extracted: first author, year of publication, country, population, sample size, study design, and source of antibiotics as well as determinants of self-medication with antibiotics. The determinants were categorised in three levels:

1. Patient level: individual characteristics of patients who use antibiotics without a medical prescription such as gender, knowledge, or preferences;
2. Healthcare professional level: characteristics and behaviour of healthcare professionals (e.g., professional knowledge about the prudent use of antibiotics), their services (e.g., provision of adequate information about the prudent use of antibiotics), and the organisation they work in (e.g., size of the pharmacy);
3. Healthcare system level: the characteristics of healthcare systems such as easy or difficult access of care for patients, payment procedures for medications or consultations, or quality of organisation (for example, monitoring systems for prescribed antibiotics).

Finally, for each determinant, whether it was from a quantitative study that used advanced statistical methods or from a descriptive/qualitative study, the association with self-medication was extracted (“positive,” “negative,” or “no association”).

### Quality Assessment

The methodological quality of the included studies was assessed using two criteria lists (one for quantitative studies and one for qualitative studies) from the “*Standard quality assessment criteria for evaluating primary research papers from a variety of fields”* handbook (12). The criteria list for the quantitative studies consisted of 14 questions and the list for the qualitative studies of 10 questions (Table A1, Supplementary Material). The questions were, among other, focused on the objective of the paper, the study design, data collection methods, and conclusions. All items on both lists were scored as “Yes” (2 points), “Partial” (1 point), “No” (0 points) or Not Applicable (the latter category only for quantitative studies). DL assessed the quality of all articles. To assess the reliability of this assessment, another researcher assessed the quality of five randomly selected studies. The quality assessments of both independent reviewers were comparable for the five reviews. As a result, further double assessment was not deemed necessary.

For each publication a score was calculated based on the sum score of all relevant items. Items not applicable to a particular study design were excluded from the calculation of the sum score. The sum score was then divided by the maximum possible score. A publication was considered as “high quality” when the quality score was in the highest quartile (>0.86 for quantitative and >1 for qualitative studies).

For each identified determinant, an indication was provided on whether it was studied in a high or low quality study. In the text, we only discuss determinants that were examined in multiple studies of which at least one is a high quality study. The remaining determinants are presented in the tables. Determinants that had no association with self-medication are not discussed in the text of this article.

## Results

### Search Results

**Figure 1:**
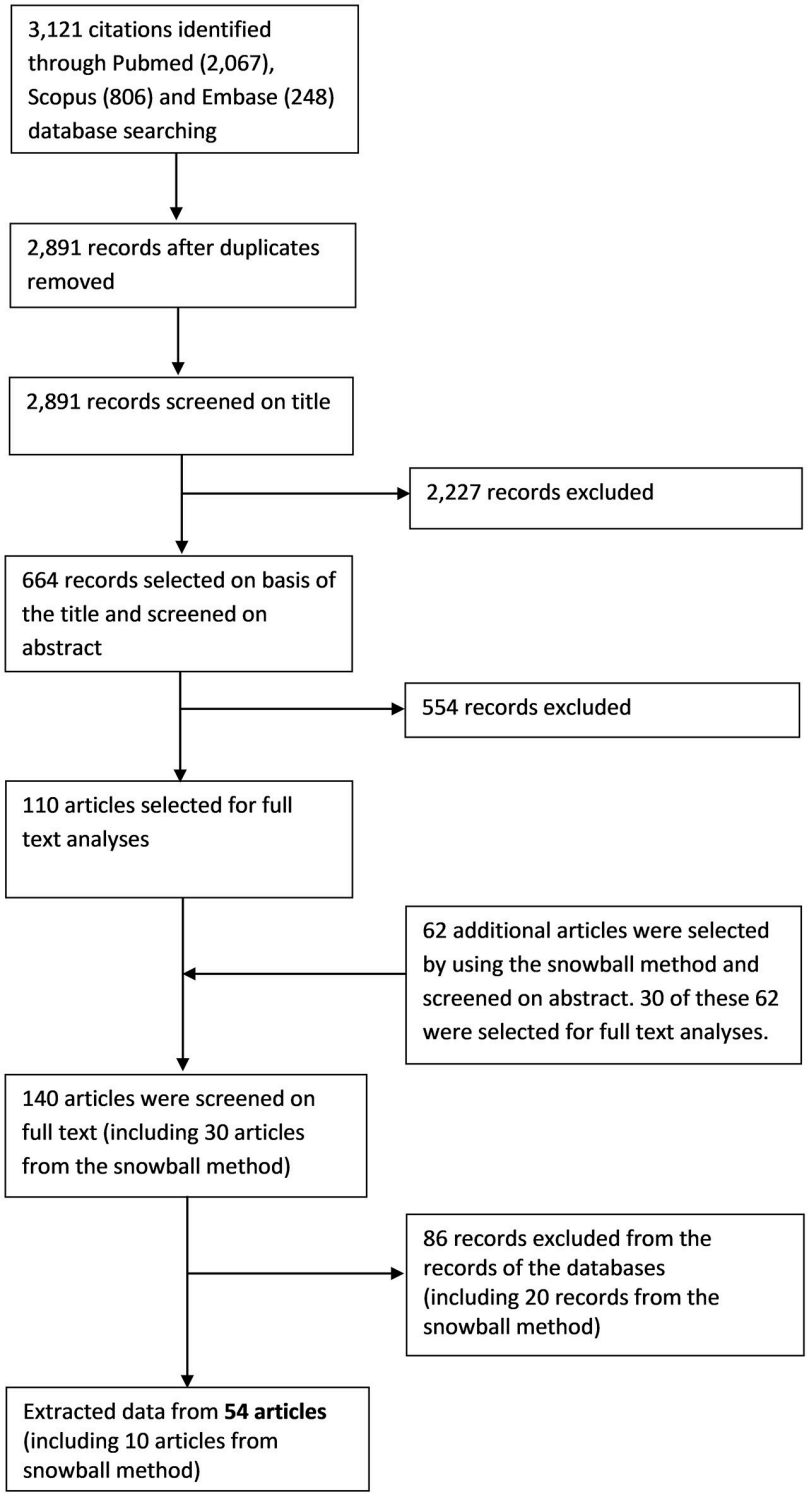
Flow diagram of the study selection process.

Our search resulted in a total of 3,121 records (2,067 records in PubMed, 806 in Scopus, 248 in Embase) of which 2,891 hits were unique. The screening of titles resulted in a total of 664 potentially relevant publications. The subsequent screening of the corresponding abstracts yielded 110 publications that were potentially relevant. After reading the full text, 44 publications were retained. By using the snowball method, 62 additional publications were regarded as potentially of interest. Of these, 10 were ultimately included, which resulted in a total of 54 publications (Figure 1) (4, 7–10, 13–61).

### Characteristics of the Included Publications

#### General Characteristics

Overall, 49 publications described quantitative and five described qualitative studies related to self-medication. The quality assessment yielded 11 quantitative (4, 9, 14, 18, 26, 29, 34, 43, 45, 48, 61) (Table A2, Supplementary Material) and one qualitative study with high quality (27) (Table A3, Supplementary Material). A total of 37 publications used patient data and 12 used pharmacist/pharmacy data. There was one publication that used both patient and pharmacist data. Two studies used data at the country level and two studies used data of the country level in combination with patient data. Overall, most studies included determinants at the patient level (*N* = 38). Eleven studies included determinants at the healthcare professional level. Two studies focused on determinants at the healthcare system level. In addition, one study focused on determinants at all three levels and two studies focused on the determinants at the patient and the healthcare system level. The majority of studies with patient data used a quantitative cross-sectional design (*N* = 30). The other seven studies were prospective observational/comparative (*N* = 3), explorative descriptive (with focus groups) (*N* = 2), quasi-experimental (*N* = 1), or an evaluation of a community-based intervention (*N* = 1). Among the studies that used pharmacist/pharmacy data, there were prospective studies (*N* = 4), cross-sectional studies (*N* = 6), a qualitative study using semi-structured questionnaires and a study that used clinical scenarios to study the behaviour and opinions of pharmacists. The study that used data from the patient and healthcare system level was qualitative and used semi-structured interviews. All four studies that used data at the healthcare level or a combination of the healthcare system level with the patient level were cross-sectional. Among the 54 studies, 41 focused on actual self-medication, seven focused (also) on intended self-medication/dispensation of antibiotics without a medical prescription and eight focused on the possession of left-over antibiotics.

#### Geographical Locations

The studies included in this review examined determinants of self-medication in 44 countries (Table A4, Supplementary Material). Of these countries 16 are located in Southern/Eastern Europe, 14 in Northern/Western Europe and 14 in (other) Anglo-Saxon and WHO Euro region countries like the former republic of Macedonia. The majority of studies were performed in Southern and Eastern Europe; most studies were performed in Spain (*N* = 14) and Italy (*N* = 9). In addition, a large number of studies were performed in the United States (*N* = 10).

### Determinants for Self-Medication

#### Patient Level

Our review showed that there was more self-medication among patients who store antibiotics at home (9, 14, 20, 42, 44, 61), who have easy access to the pharmacy and/or antibiotics (4, 15), who experience difficulties with accessing care (e.g., the physician) (15, 48, 50), who have the intention to self-medicate in the future (9, 61) and who receive advice from the pharmacist or lay persons about which antibiotics they should use (Table 1) (15, 48, 50, 53, 54). Furthermore, various studies revealed that the practice of self-medication is common among immigrants (especially Latino immigrants in the United States) (7, 30, 34, 35, 37–39). This is explained by, among others, barriers that make it difficult to use primary health care services, language barriers, and the use of imported antibiotics from their home country.

**Table 1:** Determinants of self-medication with antibiotics on the patient level.

	Association with self-medication (N of articles)^*^		
**Patient: influence on self-medication**	Positive association^**^	Negative association^**^	No association^**^
**SOCIO DEMOGRAPHIC DETERMINANTS**			
Age: middle	7 (1)	5 (3)	8 (3)
Gender: women	4 (1)	3	9 (4)
Education: higher education/longer college attendance	5 (2)	1	7 (3)
Immigrants	7 (1)	1	
Location: rural area	3 (1)	2 (1)	6 (3)
Higher working position	1 (1)		1
Parents (>1 child vs. 1 child)	1 (1)		
Parents (vs. adults)	1 (1)		
Financial barriers	1 (1)		
Being childless	1		
**TREATMENT-RELATED**			
Lack of knowledge/wrong beliefs on antibiotics	6 (1)	2 (1)	3 (1)
(Successful) treatment with (prescribed) antibiotics in the past	7 (1)		2
Keep antibiotics at home (for future needs)	6 (3)		
Use of prescribed antibiotics	2 (1)		
Dissatisfaction with healthcare system	1 (1)		
Health safety deficiencies	1 (1)		
Intended self-medication	2 (2)		
Need for antibiotics (e.g., during holiday)	2		
Quick remedies/instant fix	1		
Non-medical source of antibiotics	1		
Self-medication with NSAID	1		
**HEALTH- AND DISEASE-RELATED**			
Having a chronic disease	1	1 (1)	1 (1)
Health status: good	1 (1)	1 (1)	1
**HEALTHCARE PROFESSIONAL-RELATED**			
Received advice from pharmacist or from lay persons	5 (1)	1 (1)	
Lack of social support from healthcare professional	1 (1)		
Attended a physician in the last year	1 (1)		
Having a social contact who is working in healthcare	2		
Barriers in communication/not trusting the physician	1		
The kind of treatment and lack of information provision	1		
Not have asked about potential drug interactions	1		
**HEALTHCARE SYSTEM-RELATED**			
Lack of time/money	4 (1)		2
No (complete) reimbursement/insurance issues	2 (1)		2
Difficult access to care or healthcare professionals	3 (1)		
Easy access to/availability of antibiotics and pharmacies	2 (1)		

* *Between brackets the number of high quality studies is shown*.

** *Positive association, the determinant influences/increases self-medication; Negative association, the determinant decreases self-medication or another category (e.g., men instead of women) has a positive association; No association, no significant effect of the determinant (as revealed in quantitative studies)*.

There was support from high quality studies for more self-medication among patients with a higher position at their work (18), who are unemployed (vs. retired) (18), who have a lack of time or money (15, 20, 48, 53) and who have had a (successful) antibiotic treatment in the past and experience similar symptoms (15, 50, 53, 57). However, the low quality studies did not support the associations for these determinants (31, 44, 50). In addition, one general European study and two Southern European studies found that the previous use of prescribed antibiotics facilitated self-medication (20, 29, 54). One European study showed strong support for an association between the (previous) use of prescribed medication and self-medication with leftover antibiotics (29).

Determinants that were often studied are related to existing knowledge of patients about prudent antibiotic use and awareness of antibiotic resistance (4, 15, 20, 44, 50, 53). Most of these, mainly Southern European, studies reported that a lack of knowledge about proper antibiotic use was associated with more self-medication. There was, for instance, more self-medication among patients who are not aware of the problem of antibiotic resistance (4, 44), who believe that a prescription is not necessary for their illness (20), or who believe that the doctor would prescribe the antibiotics anyway (50, 53). However, these findings are contradicted by two (one high quality) studies from the United Kingdom and Lithuania, showing that self-medication is actually higher among patients who have good knowledge about antibiotics (43, 51).

Although there were contradictory relations for age (4, 7, 9, 14, 17–19, 21, 24, 31, 34, 42, 44–46, 48, 51, 53, 54, 59), gender (4, 7, 9, 17, 18, 21, 31, 44, 45, 48, 51, 53–55, 57, 59), the living area of the patient (4, 9, 14, 18, 23, 26, 31, 46, 49, 51, 59), and education (4, 9, 18, 20, 31, 32, 45, 48, 51, 53, 55, 57, 59), the majority of high quality studies reported no relationship between these determinants and self-medication. Contradictory results were also found for self-perceived health (the two high quality studies revealed conflicting results) (7, 18, 48) and the presence of a chronic disease (the high quality studies showed a negative or no association) (4, 18, 59).

With regard to the reimbursement patients can receive for prescribed antibiotics, contradictory results were found among the low quality studies (4, 31, 50, 53). Nonetheless, the only high quality study, that was performed within multiple European countries, showed that getting no (complete) reimbursement for prescribed antibiotics was associated with self-medication (4).

Studies demonstrated contradictory results for the association between gender (9, 31, 33, 42, 44), education (31, 42), and urban area (9, 28, 46) regarding the *possession of leftover antibiotics*, but the high quality studies did not show an association. *Intended self-medication* with antibiotics was higher among patients with a middle or younger age (9, 14, 48, 61) and among patients with a chronic disease (9, 59). With regard to the association with educational level (9, 47, 48), working position (18, 61), and living area (9, 18), the findings were contradictory (Tables A5,A6, Supplementary Material).

#### Healthcare Professional Level

Several studies from Southern and Eastern Europe have identified the shift toward equity in the patient-healthcare professional relationship as an important inducement for pharmacists to sell antibiotics OTC without a medical prescription (Table 2) (15, 20, 27, 56). This is explained by diminished respect from patients, who exert pressure on pharmacists by threatening to visit another pharmacy if they do not receive antibiotics. As many pharmacists are privately owned and do not want to lose their customers, they are inclined to dispense antibiotics without a medical prescription. Moreover, one high quality study in Romania indicated that pharmacists have the feeling the profession of being a pharmacist has been reduced to medicine salesperson and that patients treat them likewise as they perceive them to be door-to-door salesmen (27). Consistent findings were found for socially vulnerable patients: pharmacists from Romania and Portugal are more often inclined to sell antibiotics without a medical prescription to patients who are uninsured or have a low socio-economic position (27, 56). They feel these patients are in higher need of help as they cannot afford healthcare costs and costs for antibiotics on their own.

**Table 2:** Determinants of self-medication with antibiotics on the healthcare professional level.

	Association with self-medication^*^		
**Healthcare Professional**	Positive association^**^	Negative association^**^	No association^**^
**INFLUENCE ON DISPENSING ANTIBIOTICS WITHOUT A MEDICAL**			
**PRESCRIPTION BY PHARMACISTS**			
**Pharmacist characteristics**			
Professional knowledge	4		
Having the opinion that patient health comes first	1 (1)		
Insufficient knowledge about antibiotic resistance	1		
Professional attitude	1		
External responsibility (to other professionals)	1		
Indifference (lack of time to give patients an explanation about the correct antibiotics)	1		
**Relationship with patients and physicians**			
Patient pressure/diminished respect for healthcare professionals/being perceived as a salesperson	4 (1)		
Distrust in doctor's resources to carry out a good quality medical act	1 (1)		
Patient is known to have difficulty in obtaining a medical consultation	1 (1)		
Well-known patient	1		
**Pharmacy characteristics**			
Small pharmacy	2		
Pressure from the pharmacy owner	1 (1)		
Pressure of pharmaceutical companies (on chain pharmacists)	1		
**External factors**			
Social situations that affect vulnerable populations	2 (1)		
Distributors discount for antibiotics	1 (1)		
Continuing education which is focused on commercial aspects	1 (1)		
Questionable university education for pharmacists	1 (1)		
All levels of education (compared to basic, non-pharmaceutical vocational training)	1		
Dependent of working area (e.g., pharmacists would consider selling antibiotics without a medical prescription in situations where health professionals are scarce and people are in life or death situations)	1		
**INFLUENCE ON SELF-MEDICATION BY PHARMACISTS**			
Consulting a physician when feeling ill		1	
Acting according to patient leaflets	1		
Lack of continuing education on antibiotics		1	

* *Between brackets the number of high quality studies is shown*.

** *Positive association, the determinant influences/increases self-medication; Negative association, the determinant decreases self-medication or another category (e.g., men instead of women) has a positive association; No association, no significant effect of the determinant (as revealed in quantitative studies)*.

#### Healthcare System Level

In countries like Italy and Lithuania, where antibiotics are dispensed in whole packages (vs. the precise number of pills for an antibiotic course which is for instance prevalent in the Netherlands), more people are in possession of left-over antibiotics as the number of pills in the package exceeds the actual needed pills. As a result, the prevalence of self-medication is higher in these countries (Table 3) (4, 33). Findings from two other studies from Romania showed that the high prevalence of self-medication is induced by a lack of enforcement by national regulators (15, 27). When medicines regulations are not reinforced, patients are allowed to buy antibiotics without a medical prescription without any penalties for the pharmacists.

**Table 3:** Determinants of self-medication with antibiotics on the healthcare system level.

	Association with self-medication^*^		
**Healthcare System**	Positive association^**^	Negative association^**^	No association^**^
**HEALTHCARE SYSTEM CHARACTERISTICS**			
Dispensation system	1 (1)		
Unhealthy competition triggered by numerous pharmacies	1 (1)		
Professionals are working in silos	1 (1)		
Pharmacy protocols, pressure from pharmaceutical companies, informal payments	1		
Out-of-pocket expenses	1		
**REGULATION**			
Enforcement of laws	2 (1)		
Restriction of antibiotics	1		
**CULTURAL DIMENSION**			
Country Wealth	1 (1)		
Power distance	1		

* Between brackets the number of high quality studies is shown.

** *Positive association, the determinant influences/increases self-medication; Negative association, the determinant decreases self-medication or another category (e.g., men instead of women) has a positive association; No association, no significant effect of the determinant (as revealed in quantitative studies)*.

## Discussion

Self-medication with antibiotics among ambulatory care patients is a complex phenomenon that is driven by a wide variety of determinants. These determinants are related to the patient level (e.g., storage of antibiotics at home), the healthcare professional level (e.g., pressure from demanding patients to sell antibiotics without a medical prescription) and the healthcare system level (e.g., dispensing antibiotics in whole packages). Despite the broad range of national laws and legal regulations to reduce non-prudent use of antibiotics in the majority of European and Anglo-Saxon countries (62), the majority of determinants that drive patients to self-medicate are not easy to combat, making self-medication a persistent problem.

A recent EU wide study shows that EU countries focus most national laws on the healthcare system level and that sixteen EU countries dispense whole packages of antibiotics instead of precise numbers (62). Our review demonstrates that dispensing whole packages leads to a higher number of people who are in the possession of leftover antibiotics and that a broad range of determinants are related to the patient level. Therefore, although countries are putting a great deal of effort into resolving the problem of self-medication with antibiotics, they should focus on more areas and pay specific attention to the patient level. A large number of EU countries implement media campaigns to raise awareness among the population and to educate them about antibiotic use, but it is questionable whether these campaigns use the right messages and target the correct population groups. As shown in this review, not only the vulnerable population groups (e.g., immigrants or those who are unemployed) have a higher probability to self-medicate with antibiotics, but also the higher educated groups who search for convenience and an active role in their own health care. Interventions that are focused on the population should take this into account to ensure all different population groups are reached.

Determinants at the patient level, such as lack of knowledge, favourable beliefs toward self-medication and storage at home were also found to determine self-medication of non-steroidal anti-inflammatory drugs (NSAIDs) (63, 64). Moreover, studies that focused on self-medication with medicines other than antibiotics (e.g., analgesics) were also inconclusive with regard to determinants such as gender and age (65–68). However, whilst self-medication with other medicines is usually more prevalent in higher educated persons, this is not the case for antibiotics (63, 65, 67).

The focus of studies on determinants of self-medication at the patient level is not exclusive for antibiotics, but also concerns other medicines. Literature on self-medication in general, seems to neglect determinants at the healthcare professional and the healthcare system level (63–68). Among studies that aimed to explain self-medication with other medicines than antibiotics, only one explored the influence of public or private health services (66). Our review shows that future studies are required as these determinants seem vital in explaining self-medication with antibiotics.

Overall, contradictory results were found for a number of potential determinants. This may be explained by the fact that data were collected in different contexts and using different methods. For example, a Greek study showed a positive association between the level of education and self-medication (45) while a Lithuanian study did not find such an association (18). This may be explained by the fact that the Greek study was only performed in one city whilst the Lithuanian one was carried out among both urban and rural populations; in cities there are probably more opportunities to obtain antibiotics without a medical prescription. Another possible explanation for these contradictory results is that the relevance of some determinants is country dependent, which can be illustrated by other findings in this review. The relatively high use of antibiotics as self-medication among immigrants was mainly found in the United States and higher education in western European countries has a positive association with self-medication, whereas in southern European countries, there appears to be no such association. Furthermore, having less knowledge about antibiotics is associated with more self-medication in southern European countries, while in the United Kingdom patients more often self-medicate when they have more knowledge. These kinds of inconsistent findings suggest possible spurious relations; although determinants at the patient level seem to influence self-medication, there may be other, unseen high level determinants causing self-medication such as welfare status, economic conditions or existing social norms about health.

The results in our review mainly applied to Southern/Eastern European countries as the majority of studies were performed here. Northern/Western European countries were almost always included in multiple country studies which makes it unknown if the found results also apply to these countries. Still, regarding the broad variation of found determinants it is to be expected that the found determinants also play a role in Northern/Western and Anglo-Saxon countries. Considering the finding that results are often contradictory and dependent of the setting in which they were studied, more research is needed to explore determinants within the WHO Euro Region to gain insight into which determinants are country dependent and which are not.

Over the last decade, the relationship between healthcare professionals and patients has changed because of, among other, the rise of global consumerism and information available on the internet. In this review, four articles showed that the relationship has changed unfavourably for pharmacists as they experience more patient pressure (15, 20, 27, 56). Not only are patients more demanding, they also view antibiotics as easily available products that can be bought at any time. The eagerness of patients for antibiotics is even more impelled if they view antibiotics as necessary medications to cure their diseases with no or minor danger of side effects (69, 70). This is especially the case among migrant populations from, for instance, Asian or African countries (71, 72). Given the rise of global migration, the amount of patients within Europe that hold these erroneous beliefs and exert pressure on healthcare professionals to prescribe or dispense antibiotics, will increase. Although it is important to encourage more active patient participation, attention also needs to be paid to the influence of these changes on the position of healthcare professionals to preserve their important role as gatekeepers to care and antibiotics.

While the majority of studies in our review focused on determinants that explain actual self-medication, some of them also examined the influence on the intention to self-medicate (such as storage of antibiotics or the possession of leftover antibiotics) (9, 14, 18, 28, 31, 33, 42, 44, 46–48, 57, 59, 61). The determinants that have been examined to explain the intention to self-medicate are mainly in line with those that explain actual self-medication (such as the use of prescribed antibiotics or living area).

### Strengths and Limitations

As far as we know this is the first systematic review on determinants associated with self-medication with antibiotics. We were able to include a substantial number of studies examining factors at a patient level; however, the number of studies for the healthcare professional and healthcare system levels was limited. Despite our comprehensive search it is possible that we did not find all information about self-medication due to publication bias. Still, we found a wide variety of studies which focused on self-medication. Another limitation is the comparability of the studies because of the different outcome measures they presented and the variety of study designs. In order to see whether the evidence for a determinant was from a high quality study, we used the criteria lists of the “*Standard quality assessment criteria for evaluating primary research papers from a variety of fields”* handbook. These lists were developed to assess a broad range of qualitative and quantitative articles. As a result of their general nature, they are less elaborated than checklists that specifically focus on certain types of studies like the STROBE checklist for cross-sectional studies and may not have been specific enough for our study. A third limitation is that there was an overrepresentation of studies that were performed in Eastern/Southern European countries (specifically from Greece and Spain) and the United States (until now studies have been restricted to Latino immigrants). In these countries the prevalence of self-medication is higher than in the other countries included in our literature search (1). Therefore, it is possible that determinants that were found to have an association with self-medication are country-dependent and are therefore not generalizable to all European and Anglo-Saxon countries.

### Policy Implications and Future Research

This review provides an overview of the determinants that are associated with self-medication and could therefore be used to design interventions. To stimulate the prudent use of antibiotics, a multifaceted approach is recommended as the determinants for self-medication are found at different levels. Interventions and policies that simultaneously target patients and healthcare professionals will probably be more effective than single-factor approaches. For instance, by combining mass-media campaigns to increase the knowledge on antibiotics with education of healthcare professionals and a better access to healthcare (e.g., by increasing the number of general practitioners in a country). In all European countries, obtaining antibiotics without a medical prescription is already illegal (6). Yet, the enforcement of laws and regulations prohibiting the sales of antibiotics without prescription is necessary to ensure the sustainability of the aimed behavioural change. Special attention should be paid to the use of leftover antibiotics as this practice is hard to prevent with the implementation of legal measures and needs other measures such as appropriate health education of patients.

An important finding was that most studies focused on the patient perspective. While it is indeed the patient who decides to use antibiotics on his/her own initiative without consulting a doctor, healthcare professionals play an important role in influencing the behaviour of these patients, particularly pharmacists. They are key players in educating patients about the proper use of medicines as they should refer their customers to the physician before taking any medication by themselves (73, 74). This is especially important because pharmacists are usually the last contact with the patients before they receive the antibiotics (75). These interactions between patients and healthcare professionals suggest that more research is needed on the perspective of healthcare professionals. The same holds for the healthcare system level. There is still much to be explored on the role governments and other institutions can play on the prudent use of antibiotics, including self-medication. Furthermore, the influence of the pharmaceutical industry on the prescribing/dispensing behaviour of healthcare professionals and the consumption behaviour of patients, needs to be investigated. For non-EU countries like Bangladesh or India, there is evidence that pharmaceutical suppliers and medical representatives stimulate GPs to prescribe antibiotics (76, 77). In addition, a recent study which identified determinants of antibiotic dispensing without prescription, reported pressure exerted by pharmacy owners on their workers in non-EU countries to (inappropriately) dispense antibiotics and thereby increase profits (78). It is important to also understand the contribution of the pharmaceutical industry, for instance by advertising or marketing, in European and Anglo-Saxon countries to the problem of self-medication to decide whether they should also be involved in solving this issue.

## Conclusion

Self-medication with antibiotics is driven by a variety of determinants which act on different levels. To ensure the prudent use of antibiotics, appropriate multifaceted interventions are needed that target all relevant levels: the patient, healthcare professionals, and the healthcare system. In addition, future research is needed to gain more insight into the influence of determinants of self-medication at the healthcare professional and healthcare system levels in order to design effective interventions in these settings.

## Author Contributions

DL performed the literature review, screened titles, and abstracts, extracted data from the selected publications, assessed the quality of the included studies and wrote the manuscript. LvD participated in the planning of the study, screened titles, and abstracts, checked the extracted data and contributed to the manuscript. JP participated in the planning of the study, screened titles, and abstracts and contributed to the manuscript. FS participated in the planning of the study and contributed to the manuscript. All authors read and approved the final manuscript.

### Conflict of Interest Statement

The authors declare that the research was conducted in the absence of any commercial or financial relationships that could be construed as a potential conflict of interest.
