# Supplementary material for: Determinants of Self-Medication With Antibiotics in European and Anglo-Saxon Countries: A Systematic Review of the Literature
Source: Front Public Health. 2018 Dec 17;6:370. doi: 10.3389/fpubh.2018.00370 (PMC6304439; doi:10.3389/fpubh.2018.00370)
Supplement: Supplementary file 1 [file Table_1.DOCX]

Table A1: *Quality assessment lists for quantitative and qualitative studies*

| **Quality assessment list for quantitative studies** | | **Quality assessment list for qualitative studies** |
| --- | --- | --- |
| 1. | Question/objective sufficiently described? | 1. Question/objective sufficiently described? 2. Study design evident and appropriate? 3. Context for the study clear? 4. Connection to a theoretical framework/wider body of knowledge? 5. Sampling strategy described, relevant and justified? 6. Data collection methods clearly described and systematic? 7. Data analysis clearly described and systematic? 8. Use of verification procedure(s) to establish credibility 9. Conclusions supported by the results? 10. Reflexivity of the account? |
| 2. | Study design evident and appropriate? |  |
| 3. | Method of subject/comparison group selection or source of information/input variables described and appropriate? |  |
| 4. | Subject (and comparison group, if applicable) characteristics sufficiently described? |  |
| 5. | If interventional and random allocation was possible, was it described? |  |
| 6. | If interventional and blinding of investigators was possible, was it reported? |  |
| 7. | If interventional and blinding of subjects was possible, was it reported? |  |
| 8. | Outcome and (if applicable) exposure measure(s) well defined and robust to measurement/ misclassification bias? Means of assessment reported? |  |
| 9. | Sample size appropriate? |  |
| 10. | Analytic methods described/justified and appropriate? |  |
| 11. | Some estimation of variance is reported for the main results? |  |
| 12. | Controlled for confounding? |  |
| 13. | Results reported in sufficient details? |  |
| 14. | Conclusions supported by the results? |  |
