# Supplementary material for: Determinants of Self-Medication With Antibiotics in European and Anglo-Saxon Countries: A Systematic Review of the Literature
Source: Front Public Health. 2018 Dec 17;6:370. doi: 10.3389/fpubh.2018.00370 (PMC6304439; doi:10.3389/fpubh.2018.00370)
Supplement: Supplementary file 2 [file Table_2.DOCX]

Table A2: *Quality scores for the quantitative studies*

| **First author, year** | **Quality score*** |
| --- | --- |
| **Quantitative studies (max. 28 points)** |  |
| Grigoryan (2008) | 1 |
| Andrajati (2005) | 0,95 |
| Grigoryan (2006) | 0,95 |
| Landers (2010) | 0,95 |
| Napolitano (2013) | 0,95 |
| Zoorob (2016) | 0,95 |
| Berzanskyte (2006) | 0,90 |
| Edwards (2002) | 0,86 |
| Grigoryan (2007) | 0,86 |
| McNulty (2007) | 0,86 |
| Mitsi (2005) | 0,86 |
| Contopoulos-Ioannidis (2001) | 0,81 |
| Ivanovska (2013) | 0,81 |
| Mainous (2009) | 0,81 |
| Radosevic (2009)  Roque (2015)  Scaioli (2015) | 0,81  0,81  0,81 |
| Tesař (2008) | 0,81 |
| Belkina (2017) | 0,77 |
| Borg (2002) | 0,77 |
| Kardas (2007) | 0,77 |
| Mira (2014) | 0,77 |
| McNulty (2006)  Pavyde (2015)  Hu (2015) | 0,77  0,77  0,77 |
| Zapata-Cachafeiro (2014) | 0,77 |
| Aljinović-Vučić (2005) | 0,72 |
| Bernabé (2013) | 0,72 |
| Damian (2014) | 0,72 |
| Deschepper (2007) | 0,72 |
| Muras (2013) | 0,72 |
| Olczak (2006) | 0,72 |
| Papaioannidou (2009) | 0,72 |
| Di Matteo (2005) | 0,68 |
| Llor (2009) | 0,68 |
| Mainous (2005) | 0,68 |
| Marković-Peković (2012) | 0,68 |
| Matuz (2007) | 0,68 |
| Ribas (2008) | 0,68 |
| Richman (2001) | 0,68 |
| Väänänen (2006) | 0,68 |
| Blundell (2001) | 0,63 |
| Jorgji (2014) | 0,63 |
| Llor (2010) | 0,63 |
| Muscat (2006) | 0,63 |
| Simo (2012) | 0,63 |
| Boseveska (2012) | 0,54 |
| Gonzales (2006) | 0,54 |
| Plachouras (2010) | 0,50 |

*The presented sum score is the weighted score of all valid items (% of maximum score).
