# Supplementary material for: Determinants of Self-Medication With Antibiotics in European and Anglo-Saxon Countries: A Systematic Review of the Literature
Source: Front Public Health. 2018 Dec 17;6:370. doi: 10.3389/fpubh.2018.00370 (PMC6304439; doi:10.3389/fpubh.2018.00370)
Supplement: Supplementary file 3 [file Table_3.DOCX]

Table A3: Quality scores for the qualitative studies

| **First author, year** | **Quality score*** |
| --- | --- |
| **Qualitative studies (max. 20 points)** |  |
| Ghigha (2015) | 1 |
| Larson (2006) | 0,80 |
| Anghel (2013) | 0,75 |
| Mainous (2008) | 0,70 |
| Dameh (2012) | 0,65 |

*The presented quality score is the weighted score of all valid items (% of maximum score).
