# Supplementary material for: Determinants of Self-Medication With Antibiotics in European and Anglo-Saxon Countries: A Systematic Review of the Literature
Source: Front Public Health. 2018 Dec 17;6:370. doi: 10.3389/fpubh.2018.00370 (PMC6304439; doi:10.3389/fpubh.2018.00370)
Supplement: Supplementary file 4 [file Table_4.DOCX]

Table A4: *Study sites**

| **Country** | **Number of times included in single country studies** | **Number of times included in multiple country studies** |
| --- | --- | --- |
| *Southern/Eastern Europe* |  |  |
| Spain | 10 | 4 |
| Italy | 3 | 6 |
| Croatia | 1 | 6 |
| Lithuania | 2 | 5 |
| Romania | 3 | 4 |
| Czech Republic | 1 | 5 |
| Greece | 4 | 2 |
| Malta | 1 | 5 |
| Poland | 2 | 4 |
| Slovenia |  | 5 |
| Slovakia/Slovak Republic |  | 4 |
| Hungary | 1 | 2 |
| Bulgaria |  | 1 |
| Estonia |  | 1 |
| Portugal | 1 | 1 |
| Serbia |  | 1 |
|  |  |  |
| *Northern/Western Europe* |  |  |
| United Kingdom** | 1 | 3 |
| The Netherlands |  | 6 |
| Austria |  | 5 |
| Belgium |  | 5 |
| Denmark | 1 | 4 |
| Sweden | 1 | 5 |
| Ireland** |  | 4 |
| Israel |  | 4 |
| Luxembourg |  | 4 |
| Great Britain** |  | 2 |
| Finland |  | 1 |
| France |  | 1 |
| Germany |  | 1 |
| Norway |  | 1 |
|  |  |  |
| *WHO Euro region and (other) Anglo-Saxon countries* |  |  |
| United States | 9 | 1 |
| Former Yugoslav Republic of Macedonia | 2 | 1 |
| Russia | 1 | 1 |
| Albania | 1 |  |
| Australia | 1 |  |
| China |  | 1 |
| Brazil |  | 1 |
| Japan |  | 1 |
| Mexico |  | 1 |
| New Zealand | 1 |  |
| The Philippines |  | 1 |
| Republic of Srpska, Bosnia and Herzegovina | 1 |  |
| South Africa |  | 1 |
| Turkey |  | 1 |

**The number per country is based on multiple country studies or on single country studies*

***These countries also belong to the Anglo-Saxon countries*
