# Supplementary material for: Determinants of Self-Medication With Antibiotics in European and Anglo-Saxon Countries: A Systematic Review of the Literature
Source: Front Public Health. 2018 Dec 17;6:370. doi: 10.3389/fpubh.2018.00370 (PMC6304439; doi:10.3389/fpubh.2018.00370)
Supplement: Supplementary file 5 [file Table_5.DOCX]

Table A5: Determinants of intended self-medication with antibiotics on the patient level

|  | **Association with self-medication**  **(N of articles)*** | | |
| --- | --- | --- | --- |
| **Patient: influence on intended self-medication** | *Positive*  *association*** | *Negative association*** | *No association*** |
| *Socio demographic determinants* |  |  |  |
| - Age: middle and younger age | 4 (4) |  |  |
| - Adults (vs. for their children) | 1 |  |  |
| - Education: higher educational level | 2 (1) |  | 1 (1) |
| - Occupation: being unemployed | 1 (1) |  |  |
| - Higher socio-economic position | 1 (1) | 1 (1) |  |
| - Location: rural area | 1 (1) |  |  |
| *Treatment-related* |  |  |  |
| - Lack of knowledge/wrong beliefs | 1 (1) |  |  |
| - Storage of antibiotics | 1 (1) |  |  |
| - Public clinic patients (vs. private clinic patients) | 1 (1) |  |  |
| - Intake of (prescribed) antibiotics in the last year | 1 |  |  |
| *Health- and disease-related* |  |  |  |
| - Presence of a chronic disease | 2 (1) |  |  |
| - Lower self-rated health status | 1 (1) |  |  |

*Between brackets the number of high quality studies is shown

***Positive association: the determinant influences/increases self-medication; Negative association: the determinant decreases self-medication or another category (e.g. men instead of women) has a positive association; No association: no significant effect of the determinant (as revealed in quantitative studies)*
