# Supplementary material for: Determinants of Self-Medication With Antibiotics in European and Anglo-Saxon Countries: A Systematic Review of the Literature
Source: Front Public Health. 2018 Dec 17;6:370. doi: 10.3389/fpubh.2018.00370 (PMC6304439; doi:10.3389/fpubh.2018.00370)
Supplement: Supplementary file 6 [file Table_6.DOCX]

Table A6: Determinants of storage of antibiotics on the patient level

|  | **Association with self-medication**  **(N of articles)*** | | |
| --- | --- | --- | --- |
| **Patient: influence on the possession of leftovers** | *Positive*  *association*** | *Negative association*** | *No association*** |
| *Socio cultural background determinants* |  |  |  |
| - Age: middle or younger | 1 |  | 3 |
| - Gender: men | 1 |  | 4 (1) |
| - Location: urban area |  | 2 |  |
| - Medical students (versus health profession students) | 1 (1) |  |  |
| - Education: higher | 1 |  | 1 |
| - Marital status: single | 1 |  |  |
| *Treatment-related* |  |  |  |
| - Lack of knowledge about the correct use of antibiotics | 2 |  |  |
| - Intended self-medication | 1 (1) |  |  |
| - Treatment duration | 1 |  |  |
| - Having two or three daily doses vs. once-daily doses | 1 |  |  |
| - Bought antibiotics without prescription | 1 |  |  |
| - Use of liquid antibiotics | 1 |  |  |
| - Source of antibiotics was non-professional | 1 |  |  |
| - Intake of (prescribed) antibiotics in the last year | 1 |  |  |
| *Health- and disease-related* |  |  |  |
| - Health status: good | 1 |  |  |
| *Healthcare professional-related* |  |  |  |
| - Received no information (e.g. about diagnosis) | 2 |  |  |
| - Having a relative working in a health-related field | 1 |  |  |
| *Healthcare system* |  |  |  |
| - Dispensation of whole packages | 1 |  |  |

*Between brackets the number of high quality studies is shown

***Positive association: the determinant influences/increases self-medication; Negative association: the determinant decreases self-medication or another category (e.g. men instead of women) has a positive association; No association: no significant effect of the determinant (as revealed in quantitative studies)*
